# Community structure variability of Uropodina mites (Acari: Mesostigmata) in nests of the common mole, *Talpa europaea*, in Central Europe

**DOI:** 10.1007/s10493-016-0017-6

**Published:** 2016-02-09

**Authors:** Agnieszka Napierała, Anna Mądra, Kornelia Leszczyńska-Deja, Dariusz J. Gwiazdowicz, Bartłomiej Gołdyn, Jerzy Błoszyk

**Affiliations:** Department of General Zoology, Faculty of Biology, Adam Mickiewicz University, Umultowska 89, 61-614 Poznan, Poland; Natural History Collections, Faculty of Biology, Adam Mickiewicz University, Umultowska 89, 61-614 Poznan, Poland; Department of Forest Pathology, Poznań University of Life Sciences, Wojska Polskiego 71C, 60-625 Poznan, Poland; Laboratorio de Ecología Tropical Natural y Aplicada, Universidad Estatal Amazónica, Campus Principal Km 2.1/2 via a Napo (Paso Lateral), Puyo, Pastaza Ecuador

**Keywords:** Uropodina mites, Merocenoses, Nidicolous species, *Phaulodiaspis*, Nests

## Abstract

Underground nests of *Talpa europaea*, known as the common mole, are very specific microhabitats, which are also quite often inhabited by various groups of arthropods. Mites from the suborder Uropodina (Acari: Mesostigmata) are only one of them. One could expect that mole nests that are closely located are inhabited by communities of arthropods with similar species composition and structure. However, results of empirical studies clearly show that even nests which are close to each other can be different both in terms of the species composition and abundance of Uropodina communities. So far, little is known about the factors that can cause these differences. The major aim of this study was to identify factors determining species composition, abundance, and community structure of Uropodina communities in mole nests. The study is based on material collected during a long-term investigation conducted in western parts of Poland. The results indicate that the two most important factors influencing species composition and abundance of Uropodina communities in mole nests are nest-building material and depth at which nests are located. Composition of Uropodina communities in nests of moles was also compared with that of other microhabitats (e.g. rotten wood, forest litter, soil) based on data from 4421 samples collected in Poland. Communities of this habitat prove most similar to these of open areas, especially meadows, as well as some forest types.

## Introduction

The relations between soil arthropods and vertebrates formed in the long process of evolution can have a variety of forms. Three major types are (1) trophic relations (arthropods feed on vertebrate hosts or vice versa), (2) phoresy (arthropods travel on vertebrate hosts), and (3) microhabitat dependence (arthropods thrive in conditions generated by vertebrate hosts). The third type can be observed especially in some groups of mites inhabiting unstable microhabitats, such as bird nests (Błoszyk and Olszanowski 1985; Valera et al. 2003; Błoszyk et al. 2005; Gwiazdowicz et al. 2005; Błoszyk et al. 2006; Gwiazdowicz et al. 2006; Krištofík et al. 2007; Makarova et al. 2010), mammal nests (Okulova 2003; de la Fuente et al. 2004; Mašán and Stanko 2005; Oleaga et al. 2008; Gaglio et al. 2010; Mal’kova 2010), and animal dung (Cicolani 1992; Haloti et al. 2005).

The mite fauna of the common mole, *Talpa europaea* L., and its nests has been the object of research of various acarologists. Most of their studies have contributed mainly by the description of mite species new to science (amongst others, Bregetova 1956; Hyatt 1980; Mašán et al. 1994; Mašán and Fenda 2010). Because most of these species are parasites, their presence in mole nests is not surprising. However, little has been done so far to explain the occurrence of non-parasitic mites in mole nests—among the few who have attempted to account for the occurrence of such mites in mole nests are Błoszyk (1985), Mašán et al. (1994) and Błoszyk and Bajaczyk (1999).

Because nests of *T. europaea* are located underground they are very specific microhabitats. The various arthropod inhabitants can be divided into two main groups of organisms: (1) host ectoparasites (e.g. fleas, ticks, and other blood-sucking mites) and (2) commensals, which are dependent more on the habitat conditions than on the host itself.

Some studies demonstrated that Uropodina (Acari: Mesostigmata) communities in mole nests are very different from those found in other types of unstable microhabitats such as bird nests, anthills, dead wood, and animal droppings (Błoszyk and Olszanowski 1985, 1986; Błoszyk 1999; Bajerlein and Błoszyk 2004; Gwiazdowicz et al. 2005; Błoszyk et al. 2006; Gwiazdowicz et al. 2006; Majka et al. 2007; Napierała and Błoszyk 2013). One could expect that mole nests which are located in close proximity to each other are inhabited by communities of similar composition and structure. However, several empirical studies have shown that the mite fauna of such nests can be different both in terms of species composition and abundance (Błoszyk 1985).

The first accounts that make mention of differences in community composition and species abundance between Uropodina communities inhabiting nests and those living in litter, forest soil, and in open habitats can be found in Błoszyk (1999), who observed that the type of the material used for building nests is one of the pivotal factors which can have influence on species composition and abundance of mites in such habitats (Błoszyk 1985). A drawback of the previous studies is that they do not explain which environmental factors are directly responsible for these differences. The evidence presented suggested that various environmental factors—such as moisture, depth at which the nest in the soil, diameter, height, and building material of the molehill—can determine community structure of Uropodina in the mole nests. The main aim of the current study was to identify the environmental factors that influence species composition and abundance of Uropodina communities in mole nests.

### Biology of *Talpa europaea* and nest description

*Talpa europaea* occurs in Europe and Asia to western Siberia. In the north the range limit of its occurrence runs across Scotland, southern Sweden, southern Finland, and the Arctic Circle in Russia (the species does not occur in Ireland and Iceland). In the south it can be found in the Mediterranean area (with the exception of the southern parts of the Balkan Peninsula). In the western parts of Europe the species occurs in the northern part of the Iberian Peninsula (Kowalski 1971; Wąsik 2011). *Talpa europaea* has a wide distribution in Poland, as it occurs in almost the whole country, except the upper parts of the mountains in the south (Kowalski 1971; Mošanský 1974). Moles are usually found in upper layers of soil, in which they bore long tunnels, hunt for prey, and build nests (Grulich 1959; Popov 1960).

The nest chamber is usually built in high and dry places. A molehill with a nest can be 1 m high and 1–1.5 m in diameter, especially in water-logged sites. The depth at which the nest is located and the amount of soil covering it to a large extent depend on the type of soil and the groundwater level. In water-logged sites, where the level of groundwater is high, the nest chamber is usually closer to the ground surface. In places where the level of groundwater is lower, nests are located much deeper and the molehill is usually small. One molehill often comprises two or three nests, one above the other. It is quite likely that those additional nests are built in case of a sudden rise of the groundwater. In lower nests the building material is old and damp, whereas in upper nests the material is fresh and dry (Nowosad 1990). *Talpa europaea* often uses plant material for lining the nest chamber, usually parts of various plant species, depending on the availability of the material (Serafiński 1928; Stein 1950). The material used in nest constructions is uncut, just as in vole nests (*Microtus* sp.). Moreover, in mole nests there is always more building material than in nests of rodents and it is more diverse. It contains sometimes also other material, such as paper, foil, hawthorn, wild rose seeds, mammal fur, bird feathers, and corpses of dead animals (e.g. moles, voles, and polecats—*Mustela* sp.) (Nowosad 1990).

The close vicinity of mole nests is often inhabited by other small mammals, for example the European water vole (*Arvicola terrestris*). They frequently facilitate both exchange of parasites and spread of mole nest fauna. Abandoned mole nests with their tunnels are also sometimes visited by small mammals such as shrews and voles. Some mole burrows and nests are occasionally usurped by predators such as weasels, stoats, and polecats (Skuratowicz 1981).

### Mites from the suborder Uropodina as a model group

Mites from the suborder Uropodina are a well-known group in Europe. The number of European species that have been identified and described exceeds 440, which constitutes roughly 18 % of the known world Uropodina fauna (Wiśniewski and Hirschmann 1993).

Uropodina mites occur at all latitudes (except the polar regions), wherever any organic matter is accumulated. They inhabit open environments of various types, including dunes and turves on rocks in the highest parts of mountains. However, litter and soil of diverse forest areas are the most favorable habitats for them.

One of the salient characteristics of Uropodina species is their great diversity in habitat preferences. The species living in forest ecosystems constitute over 70 % of the Polish Uropodina fauna, whereas the remaining species inhabit unstable microhabitats, such as tree hollows, rotten tree trunks, anthills, bird and mammal nests, and animal feces (see e.g. Błoszyk et al. 2003; Napierała and Błoszyk 2013). Most of them are stenotopic or oligotopic, which means that they live in very specific habitats. Their dispersal abilities and reproduction strategies vary widely and often depend on the habitat in which they live (Błoszyk 1999). It has been shown in many studies that unstable microhabitats are usually populated by bisexual species, whereas soil habitats are often dominated by parthenogenetic ones, which are characterized by immense reduction of male numbers in the population (Błoszyk et al. 2004).

The deutonymphs of some species have developed the ability of passive dispersion, i.e. by means of phoresy. This is especially true for the species inhabiting unstable merocenoses. They can be carried by various groups of insects, e.g. myriapods, as well as in mammal fur and bird feathers (Gwiazdowicz 2000; Bajerlein and Błoszyk 2004; Gwiazdowicz et al. 2011).

## Materials and methods

In the past the common mole was regarded a garden and field pest and killing moles was accepted as a form of pest control. Nowadays, the common mole is a protected species in Europe and research as described here would require a permission issued by the Ethical Committee. However, our sampling predates the changes in the law regulating protection of the common mole.

The research material for this study (i.e., 210 mole nests and 116 soil samples from meadows surrounding the nests) was collected by a number of researchers in various periods and regions of Poland. However, most of the material (from 162 nests) was collected on meadows located near Jarocin (51°59′–52°04′N, 17°12′–18°17′E) in 1997–2002. The material from the examined nests was obtained by digging up the molehills. During the collection of the material the following parameters of the nests were recorded: depth of the nest location (exact to 1 cm), type of the building material, diameter of the molehill basis at its widest point (size of the molehill), height of the molehill (above ground) and moisture of the nest. The mole nests and soil samples were tightly packed into plastic bags and immediately transported to a laboratory for extraction.

The level of moisture in the building material used in the analyzed nests was estimated before placing the nest in Tullgren funnels. Each nest was placed on a sheet of paper (80 g/m^2^) for 5 min. On the basis of the marks of water left on the paper the collected nests were then divided into three groups: (1) dry nests (no visible marks of moisture on the paper), (2) slightly damp nests (visible marks of moisture on the paper but no possibility to squeeze the water out of the material) and (3) wet nests (clearly visible water drops on the paper and the water could be easily squeezed out of the material). The mesofauna was extracted with Tullgren funnels for 5–7 days and preserved in 75 % ethyl alcohol. The extracted specimens were deposited in the Natural History Collections of the Faculty of Biology at Adam Mickiewicz University in Poznań, Poland.

### Methods of description and statistical analysis

The diversity of Uropodina communities in the mole nests was estimated under scrutiny in relation to the selected ecological and environmental factors using multivariate analysis.

Canonical Correspondence Analysis (CCA) was performed to check the influence of environmental factors (soil moisture, nest-building material, depth and height of the molehill) on the composition of Uropodina communities (Ter Braak and Šmilauer 2002). The analysis included only species found in more than ten samples (n = 10) and the samples with complete information on the environmental factors (N = 140). Binary data (presence/absence) on species occurrence were used in the CCA analysis. To reduce the influence of spatial and temporal autocorrelation, data on year, day, longitude, and latitude of the samples were included into the model as co-variables. The Monte Carlo permutation test set for 5000 permutations was used to estimate the significance of particular independent variables and the whole model (Jongman et al. 1995; Ter Braak 1996). Only the variables significantly improving the model were used for the final ordination; the other factors were added to the model as supplementary variables. To check how these factors affect species diversity, a Generalized Linear Model (GLM) was generated on the basis of the CCA results, adjusting the numbering of the species in particular samples to the ordination space. The results were displayed on the CCA diagram as isolines showing the levels of species diversity (Ter Braak 1996).

A second CCA model was created to check the affinities of Uropodina species in mole nests to other habitat types, based on material collected by Błoszyk since 1992. This material consists of 4421 soil samples collected from 26 habitat types (open habitat, forests and shrubs, and merocenoses). Most samples consisted of sifted litter and soil, whereas some consisted of unsifted material (soil, litter from various swards, and wood dust from tree trunks) or material from bird, mammal, or ant nests collected from all over Poland. Information on the habitat type was introduced as explanatory variables. Monte Carlo test with 1000 permutations was performed to test the significance of the model. Because in the unimodal methods (e.g. CCA) rare species may have an unduly large influence on the calculations (Ter Braak and Šmilauer 2002), species that occurred in <50 samples were excluded from analysis.

Both CCA and GLM analyses were performed using CANOCO 4.5 software package (Ter Braak and Šmilauer 2002). The statistical significance threshold was set at α = 0.05.

## Results

### Communities of Uropodina in nests of *Talpa europaea* in Central Europe

Of the examined mole nests, 174 (83 %) were inhabited by Uropodina mites. In total we collected 7004 specimens (6892 from nests and 112 from nearby meadows), representing 25 species. The samples from the meadows served as background for comparisons with mole nests (Table 1).

**Table 1:** Uropodina species [deutonymphs (D), protonymphs (P), larvae (L), adult females (♀) and males (♂)] found in 326 samples from mole nests (210) and nearby meadows (116). Number of specimens obtained from nests (N1) and nearby meadows (N2), Number of samples (nests) in which a species occurred (S); D%, dominance; C%, co-efficient of occurrence

Species	N1	N2	S	♀	♂	D	P	L	D%	C%	Abbrev.
*Phaulodiaspis borealis* (Sellnick)^a^	3224	–	97	937	760	1403	118	6	46.8	46.2	Sp1
*Phaulodiaspis rackei* (Oudemans)	1428	–	83	438	540	364	79	7	20.7	39.5	Sp2
*Olodiscus minima* (Kramer)	670	29	43	656	1	39	3	–	9.72	20.5	Sp3
*Oodinychus karawaiewi* (Berlese)^b^	420	18	49	76	68	223	71	–	6.09	23.3	Sp4
*Nenteria breviunguiculata* (Willmann)^a^	319	39	58	93	77	182	6	–	4.63	27.6	Sp5
*Oodinychus ovalis* (CL Koch)^a^	269	1	31	73	85	99	13	–	3.9	14.8	Sp6
*Uropoda orbicularis* (Muller)^a^	134	4	31	26	–	111	1	–	1.94	14.8	Sp7
*Dinychus carinatus* (Berlese)	121	1	10	51	60	9	2	–	1.76	4.76	Sp8
*Dinychus perforatus* (Kramer)	114	2	18	31	32	45	8	–	1.65	8.57	Sp9
*Discourella modesta* (Leonardi)	96	8	20	97	–	5	2	–	1.39	9.52	Sp10
*Uroobovella obovata* (Canestrini et Berlese)	31	–	5	17	13	1	–	–	0.45	2.38	Sp11
*Pseudouropoda calcarata* (Hirschmann et Zirngiebl-Nicol)	22	–	5	7	9	5	1	–	0.32	2.38	Sp12
*Olodiscus misella* (Berlese)	12	–	1	12	–	–	–	–	0.17	0.48	Sp13
*Polyaspis patavinus* (Berlese)^a^	10	–	4	7	2	1	–	–	0.15	1.9	Sp14
*Trachytes aegrota* (CL Koch)	6	1	3	5	–	1	1	–	0.09	1.43	Sp15
*Leiodinychus orbicularis* (CL Koch)	5	–	1	5	–	–	–	–	0.07	0.48	Sp16
*Janetiella pyriformis* (Berlese)^a^	3	–	1	2	–	1	–	–	0.04	0.48	Sp17
*Uroseius hunzikeri* (Schweizer)	2	–	1	–	2	–	–	–	0.03	0.48	Sp18
*Pseudouropoda* sp.	2	–	1	1	1	–	–	–	0.03	0.48	–
*Urodiaspis tecta* (Kramer)	1	1	1	2	–	–	–	–	0.01	0.48	Sp19
*Dinychus arcuatus* (Trägårdh)	1	–	1	1	–	–	–	–	0.01	0.48	Sp20
*Dinychus inermis* (CL Koch)	1	3	1	3	1	–	–	–	0.01	0.48	Sp21
*Dinychus* sp.	1	–	1	1	–	–	–	–	0.01	0.48	–
*Urodiaspis pannonica* (Willmann)	–	1	–	1	–	–	–	–	–	–	–
*Neodiscopoma splendida* (Kramer)	–	1	–	–	1	–	–	–	–	–	–
*Cilliba cassideasimilis* Błoszyk et al.	–	2	–	–	1	1	–	–	–	–	Sp22
*Protodinychus punctatus* (Evans)	–	1	–	1	–	–	–	–	–	–	–
Total	6892	112	–	2543	1653	2490	305	13	100	–	–

D% = 100 × n/N, where *n* is the number of specimens of studied species present in collected samples and *N* is the total number of collected specimens. C% = 100 × c/C, where *c* is the number of samples in which a species was present and *C* is the total number of samples

**Fig. 1:**
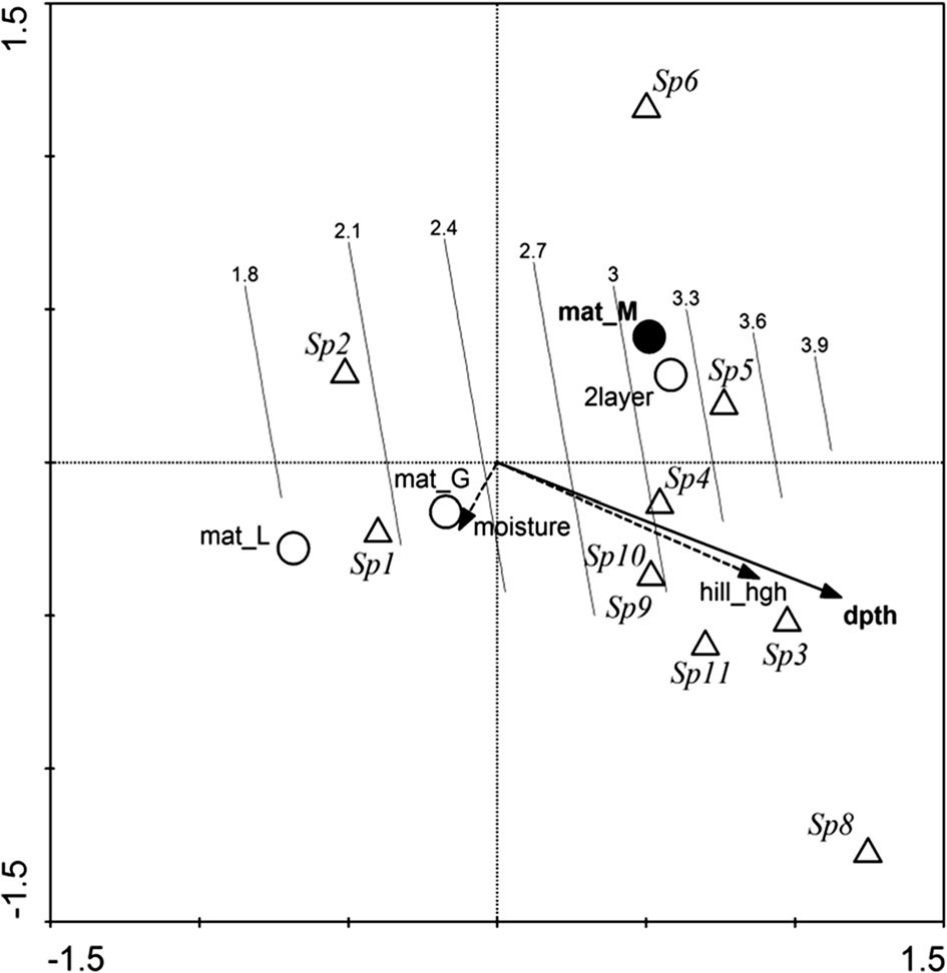
CCA diagram showing the influence of environmental factors on the composition of Uropodina communities in mole nests. Only the species found in more than ten samples (*triangles*) were included in the analysis. The *circles* and *arrows* represent environmental variables (*solid*—significant; *open circles* or *dashed arrows*—insignificant according to the Monte Carlo test, but included into the final analysis as supplementary variables). The isolines represent the species diversity fitted to the ordination space with GLM. Mat_L, nests composed of leaves; mat_G, of grass; mat_M, mixed material; dpth, location depth of the nest; hill_hgh, height of the mole hill; 2 layer, nests composed of two layers of material; moisture, nest moisture. See Table 1 for explanation of the species names

**Fig. 2:**
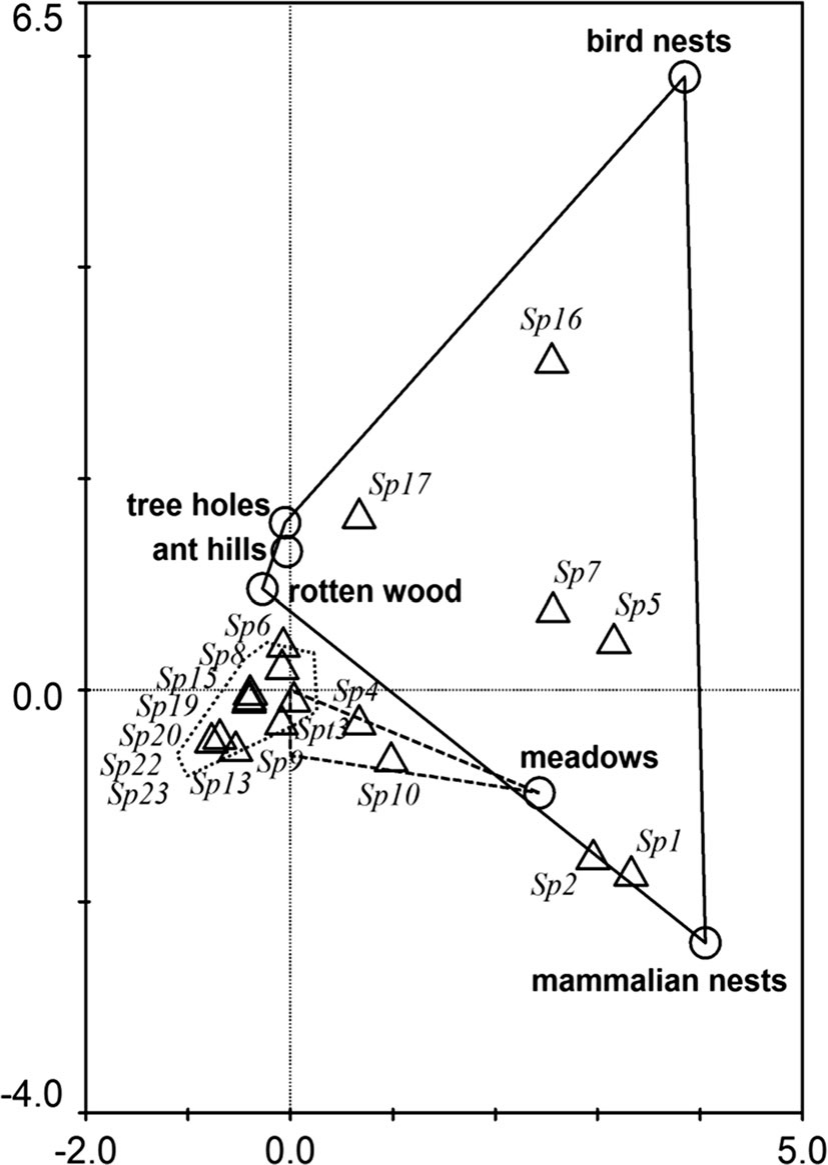
CCA diagram showing the affinities of Uropodina species found in mole nests to nearby habitat types. The *solid line* envelopes merocenoses, the *dashed line* open habitats, and the *dotted line* forest habitats. *Triangles*, species; *circles*, particular habitat types. To make the diagram transparent, only names of merocenoses and one open habitat are shown. See Table 1 for explanation of the species names

Abbreviations used on the canonical diagrams (Figs. 1, 2)

^a^Phoretic on insect

^b^Phoretic on mole fur

The domination structure of Uropodina in the mole nests was typical of unstable microenvironments. The community was dominated by two species, constituting 67 % of all representatives. One of those species (which occurs mainly in mole nests) was *Phaulodiaspis borealis*, constituting 47 % of the whole community. The second most numerous species was *Phaulodiaspis rackei*, with 20.7 %. These species were not found in the soil samples taken from the ground nearby the examined mole nests. Another nidicolous species occurring in the nests was *Uroseius hunzikeri*.

### Variation in community structure of Uropodina

The CCA indicated that building material and depth of the nests are the two most important factors for the composition of the Uropodina fauna in mole nests (Fig. 1; Table 2). The nests were mainly made out of dry grass, leaves or mixed material. The mite communities inhabiting the nests made out of mixed material were different from those found in other types of nests (λ_A_ = 0.04; F = 3.532; *p* = 0.0066). Moreover, nests built with mixed material had the highest diversity of Uropodina mites (Fig. 1). The highest average number of Uropodina was found in the nests built with grass (Table 3). In nests with leaves the number of Uropodina was rather sparse. The nests with mixed material were characterized by intermediate values (Table 3).

**Table 2:** Collation of the CCA results indicating influence of environmental factors on composition of Uropodina communities

Axes	1	2	3	4	Total inertia
Eigenvalues	0.1000	0.016	0.296	0.226	1.294
Species environment correlations	0.584	0.273	0.000	0.000	
Cumulative % variance of species data	10.2	11.7	41.7	64.6	
Cumulative % variance of species environment relation	86.5	100	0.0	0.0	
Sum of all eigenvalues					0.988
Sum of canonical eigenvalues					0.116

**Table 3:** Uropodina species in the studied mole nests with various types of building material (mean ± SD number of specimens per nest)

Species	Mole nest building material		
	Grass	Leaf	Mixed
*P. patavinus*	0.06 ± 0.38	–	–
*Dis. modesta*	0.47 ± 1.74	–	0.58 ± 2.86
*Oo. ovalis*	0.29 ± 1.29	0.41 ± 0.92	4.95 ± 17.63
*Oo. karawaiewi*	3.16 ± 8.15	0.22 ± 0.60	4.83 ± 12.02
*P. calcarata*	0.01 ± 0.12	1.84 ± 5.13	–
*O. minima*	1.30 ± 5.55	–	0.13 ± 0.34
*Ur. obovata*	–	–	0.04 ± 0.20
*Ph. rackei*	10.16 ± 27.90	3.28 ± 7.40	0.92 ± 1.72
*Ph. borealis*	27.82 ± 47.03	1.68 ± 4.20	18.08 ± 25.22
*U. orbicularis*	0.93 ± 2.64	0.39 ± 0.67	1.33 ± 4.75
*N. breviunguiculata*	1.59 ± 3.59	1.73 ± 2.73	1.38 ± 4.57
*D. perforatus*	0.15 ± 0.65	0.11 ± 0.30	0.29 ± 0.62
*D. carinatus*	0.50 ± 2.60	–	0.12 ± 0.45
*D. arcuatus*	0.01 ± 0.12	–	–
Uropodina	46.69 ± 61.12	9.18 ± 16.20	32.67 ± 42.98

The second important factor influencing the community composition was the depth at which the nests were located (λ_A_ = 0.08; F = 6.899; *p* = 0.0002). Deeper nests were usually inhabited by more species. Some species displayed a clear habitat preference for nest depth and this factor is responsible for occurrence and number of the most species. Nests close to the soil surface, usually made out of leaves and grass, had relatively uniform species composition, with a few species that were not constant across this class of nests, and in such cases the communities were dominated by *Ph. rackei* and *Ph. borealis* (Fig. 1).

Also the GLM indicated that species diversity increased with depth of the nest—nests close to the soil surface (made of leaves and/or grass) had lower species diversity than deeper nests (made of mixed material) (null deviance = 79.74; deviance = 67.95; F = 6.95; AIC = 73.040; *p* = 0.0016).

Other environmental factors, such as height of the molehill, nest moisture and multiple layers of the nest padding, seem to have had little bearing on the model (F < 1.4; *p* > 0.2). Significance of the whole model was high and reached F = 5.323; *p* < 0.001.

### Affinities of Uropodina species to other habitat types

The second CCA showed that mite communities characteristic for merocenoses of various kinds (such as mole nests) are the most variable, they also showed one of the least affinities to other habitat types (Fig. 2). *Phaulodiaspis borealis* and *Ph. rackei* are the species most affined to the mammalian nests and communities of this habitat are the most similar to these of open areas, especially meadows. Among other merocenoses, rotten wood, anthills and tree holes host the most comparable communities to these occurring in forests. Bird nests are the most separate with respect to Uropodina species composition, but share some overlap with the communities of rotten wood, anthills and tree holes, particularly the occurrence of *L. orbicularis*. The majority of Uropodina species found in mole nests prefers litter and soil of various types of forests and open habitats. In the merocenoses studied they seem to be accidental species. The model was significant at F = 28.330, *p* < 0.001.

## Discussion

The occurrence of nidicolous species, such as *Ph. rackei*, *Ph. borealis* and *Uroseius hunzikeri*, seems to be strongly associated with mole nests. These species occur mainly in nests of small mammals (Błoszyk et al. 2003). *U. hunzikeri* is a rare species in Europe; so far, it was found in Poland only in one mole nest and in one nest of a white stork (Błoszyk 1985). Mole nests are a good example of a merocenose with clear species domination structure, usually with two dominant species (e.g. *Ph. borealis* and *Ph. rackei*). The data presented in this study seem to support that Uropodina communities in unstable microhabitats are often dominated by one or two species, which constitute more than 50 % of all specimens inhabiting a given merocenose (Napierała and Błoszyk 2013). Furthermore, the results seem to follow geometric series of species-abundance curves that has been widely used to describe communities of early succession, disturbances or poor habitats (He and Tang 2008). The results obtained by Błoszyk (1985) are quite consistent with the current study. Błoszyk (1985) found as many as 18 species of Uropodina with two dominant species, viz. *Olodiscus minima* and *Ph. rackei*. More recently, Napierała and Błoszyk (2013) found 11 species of Uropodina in common mole nests, with *Ph. borealis* and *Ph. rackei* the two most frequent and dominant species.

Similar results are also given in other studies. For example, in ten nests of the common mole examined in winter by Mašán et al. (1994) they found ten species of Uropodina. *Phaulodiaspis rackei* occurred in all mole nests and was the dominant species among the Uropodina. The species also exhibited one of the highest infestation intensity among the mesostigmatid mites (i.e. the average number of individuals in the mole nests was 25.2).

Apparently, the colonization of mole nests proceeds in various possible ways. Most species probably get into mole nests directly from the adjacent areas. However, some mites presumably utilize other organisms as carrier. For example, *Oodinychus karawaiewi* was found in mole fur (Błoszyk, unpubl.). Moreover, *Ph. borealis* often uses fleas from the genus *Ctenophthalmus* inhabiting mole nests to get into a new habitat. This mite displays a strong preference for *C. assimilis*, which is usually more abundant and can be associated with a much broader spectrum of hosts (Błoszyk and Bajaczyk 1999). Little is known about other organisms that may serve as carrier for *Ph. rackei*, another common nidicolous species. A different likely carrier of these mites is the bumblebee (*Bombus* sp.), as specimens of *Ph. rackei* were also found in bumblebee nests (Mašán 2001).

The multivariate analysis indicated that the abundance of Uropodina mites to a large extent depends on the building material of the mole nest and on its depth. The nests located close to the ground surface usually contained leaves or grass, whereas those located deeper were often made of mixed organic material. The material used to build the nest certainly depends on the surrounding environment. However, each type of nest-building material creates a different microclimate which undoubtedly has a large impact on nidicolous mites. Nests made out of grass material had the highest number of Uropodina, much higher than the other nest types, which seems to corroborate Błoszyk’s (1985) observations.

The second most important factor influencing the structure of Uropodina communities is the depth of mole nests. Mites were more diverse in the deeper nests. Deeper nests are usually characterized by minor microclimatic fluctuations during the whole year, which is quite favorable for stenotopic species. On the other hand, nest located closer to the ground surface are often characterized by almost direct influence of external climatic factors (e.g. temperature and precipitation), which obviously causes unstable microclimatic conditions inside the nest. Such habitats are often visited by soil species with higher ecological tolerance; however, they usually do not form stable communities.
